# Storm-Drain and Manhole Detection Using the RetinaNet Method

**DOI:** 10.3390/s20164450

**Published:** 2020-08-10

**Authors:** Anderson Santos, José Marcato Junior, Jonathan de Andrade Silva, Rodrigo Pereira, Daniel Matos, Geazy Menezes, Leandro Higa, Anette Eltner, Ana Paula Ramos, Lucas Osco, Wesley Gonçalves

**Affiliations:** 1 Faculty of Computer Science, Federal University of Mato Grosso do Sul, Campo Grande 79070900, MS, Brazil; anderson.asantos3@gmail.com (A.S.); jonathan.andrade@ufms.br (J.d.A.S.); geazyme01@gmail.com (G.M.); leandro.t.higa@gmail.com (L.H.); wesley.goncalves@ufms.br (W.G.); 2 Faculty of Engineering, Architecture and Urbanism and Geography, Federal University of Mato Grosso do Sul, Campo Grande 79070900, MS, Brazil; rodrigoeamb@gmail.com (R.P.); daniel.matos@ufms.br (D.M.); 3 Institute of Photogrammetry and Remote Sensing, Technische Universität Dresden, 01062 Dresden, Germany; anette.eltner@tu-dresden.de; 4 Graduate Program of Environment and Regional Development, University of Western São Paulo, Presidente Prudente 19067175, Brazil; anaramos@unoeste.br (A.P.R.); lucasosco@unoeste.br (L.O.)

**Keywords:** convolutional neural network, object detection, urban floods mapping

## Abstract

As key-components of the urban-drainage system, storm-drains and manholes are essential to the hydrological modeling of urban basins. Accurately mapping of these objects can help to improve the storm-drain systems for the prevention and mitigation of urban floods. Novel Deep Learning (DL) methods have been proposed to aid the mapping of these urban features. The main aim of this paper is to evaluate the state-of-the-art object detection method RetinaNet to identify storm-drain and manhole in urban areas in street-level RGB images. The experimental assessment was performed using 297 mobile mapping images captured in 2019 in the streets in six regions in Campo Grande city, located in Mato Grosso do Sul state, Brazil. Two configurations of training, validation, and test images were considered. ResNet-50 and ResNet-101 were adopted in the experimental assessment as the two distinct feature extractor networks (i.e., backbones) for the RetinaNet method. The results were compared with the Faster R-CNN method. The results showed a higher detection accuracy when using RetinaNet with ResNet-50. In conclusion, the assessed DL method is adequate to detect storm-drain and manhole from mobile mapping RGB images, outperforming the Faster R-CNN method. The labeled dataset used in this study is available for future research.

## 1. Introduction

According to the United Nations Office for Disaster Risk Reduction [1], floods were the most common type of natural disaster in the world for the period 1998–2017, affecting 2 billion people, causing 142,088 deaths and economic losses estimated at $656 billion. In this context, also urban floods need to be considered; according to the World Urbanization Prospects [2], 36.8% of the 633 largest cities in the world are exposed to flood risk, impacting over 660 million inhabitants. An increase in urban flood risks is expected due to climate change, as an intensification of extreme events of precipitation is predicted, potentially leading to a larger water intake into an urban basin [3]. Furthermore, according to [4], changes in land use are another main factor responsible for modifying the hydrological characteristics of urban basins due to the reduction of infiltration capacities and increased runoff. Thus, urbanization leads to increased flood risk because of the impervious surfaces in urban areas [3,5]. Municipalities adopt storm-drain networks to decrease the runoff rate from extreme events and impervious surfaces and thus reduce the impacts by urban floods [6]. One way to assess urban flood risks is to model the drainage system for these watersheds at specific hydrological conditions, and thus adapt the storm-drain network to mitigate the potential damage caused by such floods. It is an essential tool for the planning and management of storm-drain system infrastructures of urban watersheds [7]. Models, such as HEC-1 and Storm Water Management Model (SWMN), evaluate the interation between rainwater and drainage system. Inputs to these models include the size, quantity, and spatial distribution of storm-drains. However, municipal management does not always possess this data, especially in developing countries.

Various remote sensing approaches have been developed to find manholes and storm-drains in urban areas automatically. For instance, [8,9] tested the usage of laser scanning (LiDAR) data. However, when compared to image-based methods, LiDAR data are expensive in terms of equipment and computational costs. Therefore, another focus has been on machine learning algorithms applied to imagery because they can be a useful and robust form to analyze data [10]. These algorithms are widely combined with computer vision techniques to process image data [11,12]. For manhole detection in aerial images, different algorithms were designed with shallow structures [13,14,15,16,17], which need a careful feature extraction method involving pre-processing steps and classification algorithms to achieve good accuracy rates [18,19]. For example, in [15], the authors achieved manhole detection accuracies of 58%. Due to the variety of images datasets (with different illumination conditions, occlusions, noise, and scale), traditional machine learning methods have a low probability of being successful to detect manhole and storm-drain, especially in high dimensionality feature space. More recent, Deep Learning (DL) based-methods have shown higher performances in computer vision tasks because they can extract features while jointly performing classification (end-to-end learning) [18].

DL methods have been successfully used to object detection [20] in several applications, such as agriculture and environmental studies [21,22], urban infrastructure [23] and health analysis [24]. Thus far, solely few works have been developed to detect manholes using DL ([25] and [26]). Reference [25] perform manhole detection in aerial images. However, according to [26], there are two main limitations for using aerial images to detect manholes: (i) The images present resolutions of about 5–10 cm/pixel, which can be insufficient to identify details of the objects, and (ii) manholes can be hidden by trees and vehicles in these images. Therefore, in [26] the authors aimed to detect manhole and storm-drains in images captured from Google Street View API. They demonstrated that street-level imagery can provide useful information to identify obstructed objects, which were not appropriately detected in aerial images.

In this paper, the state-of-the-art DL method RetinaNet was investigated to automatically detect storm-drain and manhole covers in street-level images collected with a car-mounted camera. As an additional contribution, an analyzes of the influence of different feature extractor networks (i.e., backbones) was conducted at the detection accuracy of storm-drain and manhole different from [26], which used a Faster R-CNN architecture (two-stage network) with Resnet 101 as the backbone. The one-stage network RetinaNet was chosen as the network architecture because of its state-of-the-art performance in object detection tasks [27,28,29]. Furthermore, one-stage methodologies have lower computational processing costs than two-stage approaches [20,30]. One-stage methods typically use the VGG and ResNet as network backbone [31,32], which have shown good results even compared to the DenseNet backbone [23]. ResNet backbones (ResNet-50 and ResNet-101) are used to analyze the effect of their depth on the RetinaNet classification model. Another contribution is to make the labeled dataset publicly available to allow for further DL training in this object detection application. In summary, here are the main contributions:

- The state-of-the-art DL method RetinaNet is investigated to detect Storm-drain and Manhole;
- RetinaNet is compared to Faster R-CNN, which was used for the same purpose in previous research;
- ResNet-50 and ResNet-101 backbones were assessed and;
- The data set is publicly provided for future investigations in https://sites.google.com/view/geomatics-and-computer-vision/home/datasets.

This paper is organized as followed. In Section 2, materials and methods adopted in the study are described. Section 3 presents and discusses the results obtained in the experimental analysis, and Section 4 highlights the main conclusions.

## 2. Material and Methods

To achieve the aim of this work, initially terrestrial images were acquired in the streets of the Campo Grande city (Section 2.1). The image dataset is described with details in Section 2.2, including the organization in training, validation, and testing sets. The assessed object detection methods are presented in Section 2.3. Finnaly, the assessment metrics are presented in Section 2.4. The procedure steps are the same adopted in our previous work [22].

### 2.1. Study Area

**Figure 1:**
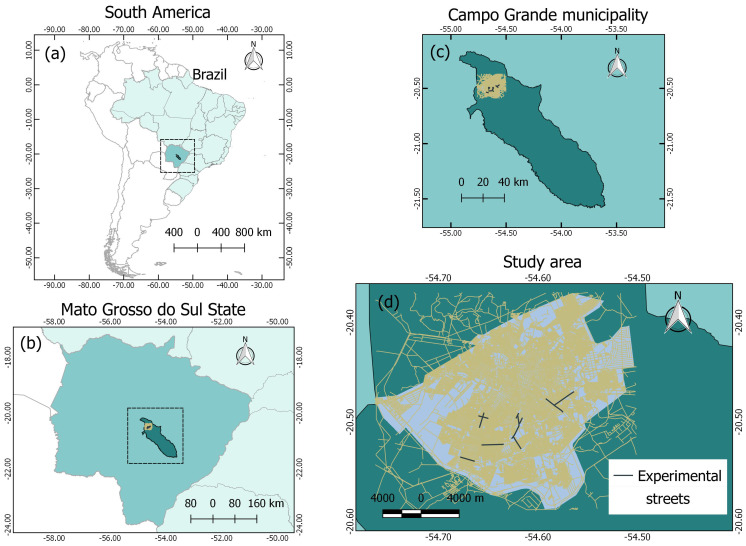
Study area in (**a**) South America and Brazil, (**b**) Mato Grosso do Sul, (**c**) Campo Grande, and (**d**) experimental streets. The black lines represent the streets used in the experiments

The images were acquired in the streets of the Campo Grande city, in the state of Mato Grosso do Sul, Brazil (Figure 1). Several damages related to floods occurred in Campo Grande in the previous years, showing a real need for detailed hydrological modeling in its urban area. Accurately mapping storm-drains and manholes is a crucial step to contribute to this modeling. The black lines in Figure 1d highlight the streets considered in our experiments.

### 2.2. Image Dataset

**Figure 2:**
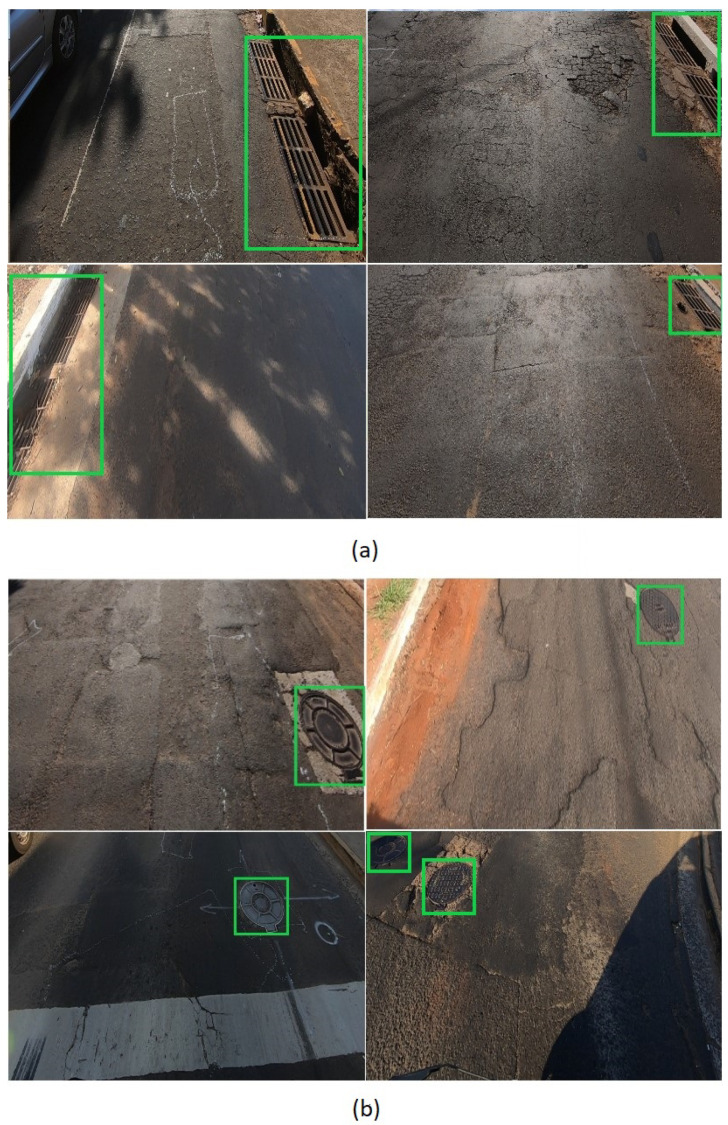
Eight examples of images containing (**a**) storm-drains and (**b**) manhole, both highlighted by green rectangles.

Storm-drain and manhole samples are presented in Figure 2, showing that the images of the dataset possess variability in terms of appearance, position, scale, and illumination. The dataset is composed of 297 images with resolutions of 1280 × 720 pixels acquired with a GoPro HERO6 Black RGB camera. This data set contains 166 manhole and 142 storm-drain objects. These images correspond to different regions of Campo Grande city. The images were cropped at 50% of the original width to remove the sky, as done by [26] and [25], resulting in images with resolutions of 1280 × 369 pixels.

The images were manually annotated by marking the manhole and storm-drains objects with rectangles (bounding boxes) and labeling each rectangle to its corresponding class. Afterward, these images were divided into two groups of training, validation, and testing sets. The first group (named 76-12-12) has 76%, 12%, and 12%, respectively, for training, validation and testing sets. The second group (named 66-15-19) has 66% of training images, 15% of validation images, and 19% of testing images. These two groups were considered to assess the methods not only in one scenario, contributing to a more robust evaluation.

Images for training, validation, and test are from different regions of the city. The idea is to avoid similarity between images from validation and test sets with the training set images to achieve a well generalizing detection model. In Table 1, the main features of our data set summarized.

### 2.3. Object Detection Method

For this study, the RetinaNet object detection method [33] was adopted. RetinaNet is a one-stage object detection method that considers class imbalance by reducing the loss assigned to images that are well-classified. Class imbalance happens when the number of background examples is larger than the examples of the object of interest, which, in this case, are storm-drains and manholes.

The training step focuses on hard-to-detect examples. RetinaNet architecture is composed of a backbone and two task-specific subnetworks. We adopted the ResNet-50 and ResNet-101 as the backbone and combined it with the Feature Pyramidal Network (FPN) [34], which represents objects at multiple scales that share high and low-level features. Two subnets are applied to the backbone’s output to perform the classification and regression tasks.

The models’ weights were initialized with weights from the same architecture pre-trained on the MS Coco dataset [35] to reduce the training time. We used the source code available on the Detectron2 toolbox [36] for our implementation. The model was trained and tested on a desktop computer with an Intel(R) Xeon(R) CPU E3-1270@3.80GHz, 64 GB memory, and an NVIDIA Titan V Graphics Card (5120 Compute Unified Device Architecture (CUDA) cores and 12 GB graphics memory) on the Ubuntu 18.04 operating system.

A learning rate of 0.01 was adopted. The number of iterations was set to 10,000 (as set in [25]). Moreover, a batch size of 4 images and 128 regions of interests was chosen for the RetinaNet and Faster R-CNN [37] methods. The results between both methods were compared because previous work on storm-drain and manhole detection [26] considered Faster R-CNN.

### 2.4. Method Assessment

The performance of RetinaNet was assessed by precision–recall curves and the average precision (AP) as adopted in [22]. To estimate the precision and recall, the Intersection over Union (IoU) was calculated. This metric is given by overlapping the area between the predicted and the ground truth bounding boxes divided by the area of union between them. Following well-known competitions in the object detection scene, a correct detection (True Positive, TP) was also considered for IoU ≥0.5, and a wrong detection (False Positive, FP) for IoU <0.5. A False Negative (FN) is assigned when no corresponding ground truth is detected. Based on the above metrics, precision (*P*) and recall (*R*) are estimated using Equations (1) and (2), respectively. The average precision is estimated by the area under the precision–recall curve.

(1) $$\begin{array}{c} P=\frac{TP}{TP+FP} \end{array}$$

(2) $$\begin{array}{c} R=\frac{TP}{TP+FN} \end{array}$$

## 3. Results and Discussions

### 3.1. Learning Results of the Object Detection Method

**Figure 3:**
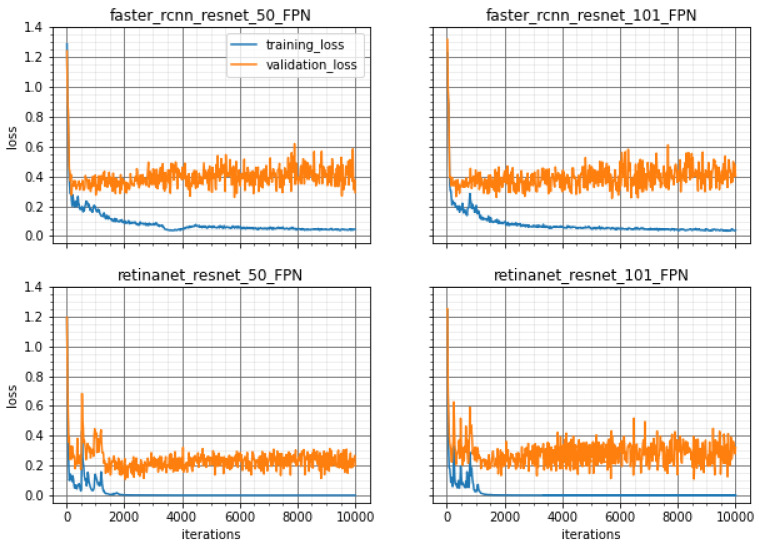
Training and Validation Loss values for all methods to the division 76-12-12 over 10,000 iterations of training model.

**Figure 4:**
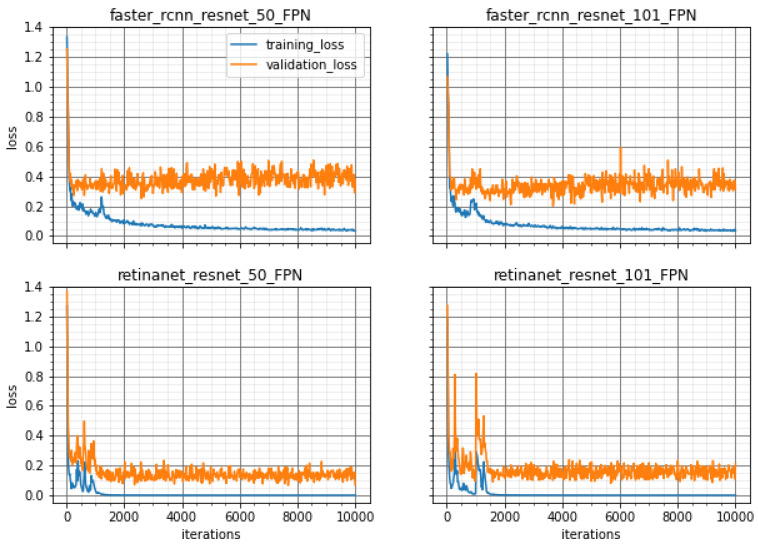
Training and Validation Loss values for all methods to the division 66-15-19 over 10,000 iterations of training model.

The training of the methods was performed with different backbones and the loss curves are shown in Figure 3 and Figure 4 for both groups, 76-12-12 and 66-12-19, respectively. These loss curves indicate that no overfitting occurred because the loss values for training and validation were similar and did not increase. Furthermore, the RetinaNet model converged at approximately 2000 iterations while the Faster R-CNN needed about 8000 iterations until the training loss curve remained flat. This was noted for both proposed divisions of training, validation, and testing sets.

### 3.2. Inference Results of the Object Detection Method

**Figure 5:**
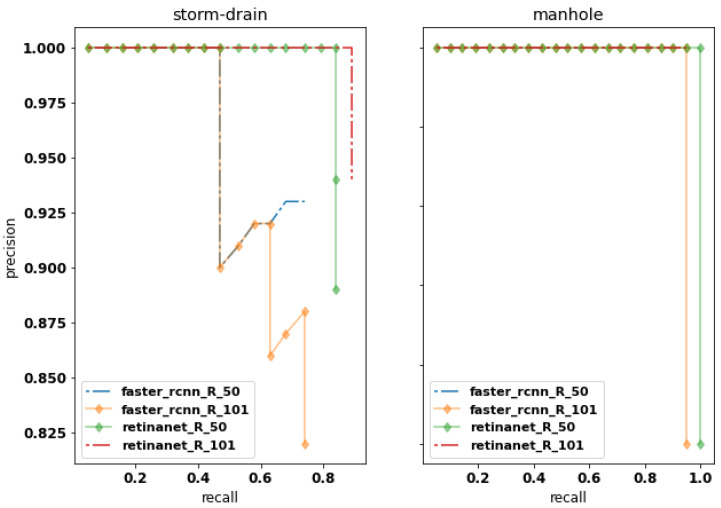
Precision–recall curves for all methods (R_50 and R_101 means ResNet-50 and ResNet-101, respectively) to the division 76-12-12. on IoU threshold at 0.5 (AP50).

**Figure 6:**
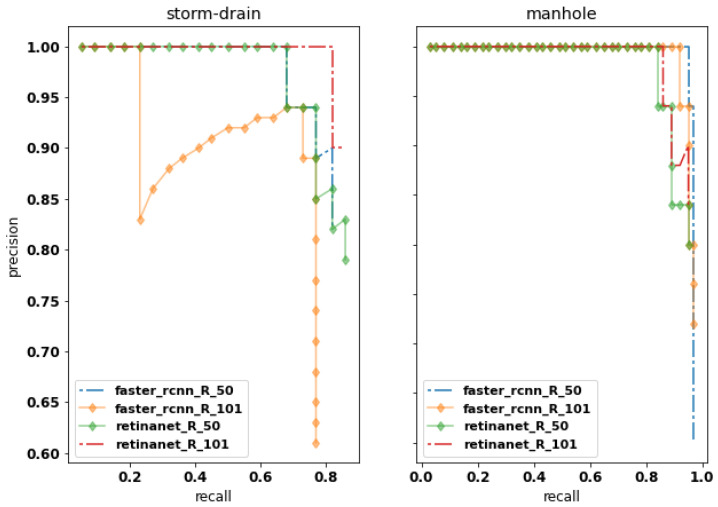
Precision–recall curves for all methods (R_50 and R_101 means ResNet-50 and ResNet-101, respectively) to the division 66-15-19 on IoU threshold at 0.5 (AP50).

The average precision (AP, %) and its mean values (mAP, %) obtained from the area under the curve are illustrated in Figure 5 and Figure 6 and in Table 2. The results on Table 2 display the IoU cutoff at 0.5 (AP50) and the AP values to each class, manhole (${\mathrm{AP}}_{mh}$) and storm-drain (${\mathrm{AP}}_{sd}$). The best AP50 values are achieved by RetinaNet, compared to Faster R-CNN, for both datasets division (76-12-12 and 66-12-19). Furthermore, RetinaNet provides the best results for the storm-drain class, which is more challenging to identify when compared to the manhole class.

**Figure 7:**
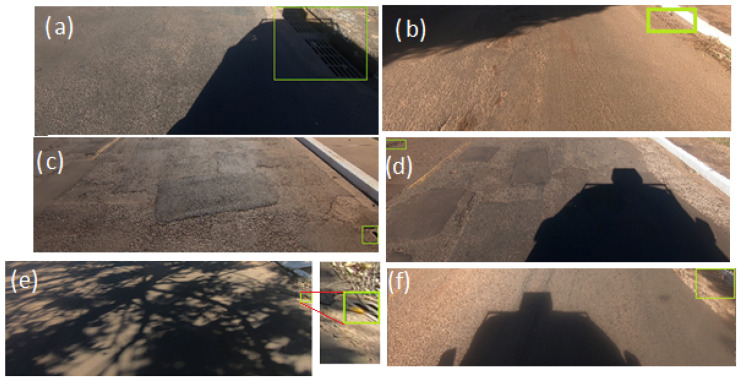
Examples of images that some models did not predict the bounding boxes to the division 76-12-12 considering (**a**) shadow presence, (**b**) small size objects, (**c**) small size objects truncated, (**d**) small size objects, (**e**) small size objects with shadow presence and (**f**) shadow presence.

Considering the images in Figure 7 it becomes obvious that not all predictions were made correctly by RetinaNet and Faster-RCNN. We found six situations of FNs (false-negative) for the division 76-12-12: Faster-RCNN (ResNet-101) achieved four FNs (Figure 7b–f); Faster-RCNN (ResNet-50 ) not only achieved the same FNs, but also did not detect the object of interest in Figure 7a; RetinaNet (ResNet50) and RetinaNet (ResNet101) provided only two FNs each one. The objects were not detected in Figure 7b,e when using RetinaNet (ResNet-50), while RetinaNet (ResNet-101) did not detect them in Figure 7c,d. These images were challenging for the trained network due to illumination and noise conditions (Figure 7f). Nevertheless, even in these conditions RetinaNet (ResNet-50) achieved an IoU value of 0.77 with a corresponding confidence (score) value of 0.99.

To examine the importance of our proposed framework, a discussion is presented with a selection of similar studies. A study by [38] achieved an F1-measure score of 0.95 using mobile laser scanning data and a random forest model to identify manholes. The approach, although showing high performance for a shallow learning method, is more expensive regarding data acquisition than RGB data imagery. Another approach by [25] detected manholes in aerial imagery with an accuracy of 99% and a positioning error below 0.7 m. In that study, a Single Shot multi-box Detector (SSD) method was developed and evaluated for images mostly captured from the nadir position. A paper by [25] evaluated different DL networks to detect manholes similar to the current study. However, they utilized aerial images. Their method faced the same conditions as the study by [25]; the high-resolution imagery from the nadir position returned lower accuracies (ranging from 0.67 to 0.89) than our approach. However, it is difficult to compare the results with the performance of our method because they evaluated images from a different point-of-view. The investigated DL-based approach identified hard-to-detect instances with proximal accuracy metrics, in different sizes, point-of-view, and positions, which demonstrates its versatility.

Based on the qualitative and quantitative analysis, RetinaNet outperformed Faster-RCNN, mainly due to more reliable detection in challenging situations. It is important to highlight that the RetinaNet method focuses on hard-to-detect examples in the training task. Furthermore, a higher performance was revealed for manhole detection compared to storm-drain, which confirms the previous work by [26]. Furthermore, only small differences was verified in the results obtained with different backbones. According to [26], results from deep models (like ResNet101) could deteriorate the detection’s quality when using aerial images because the last layers of the model are not able to respond to too small objects, as shown in [25]. Thus, street-level images can provide a good alternative to detect manhole and storm-drain objects in images.

Previous work [22,39] showed the potential of RetinaNet in other remote sensing applications, which was also verified in the detection of manhole and storm-drain. However, additional experiments are still necessary to evaluate its effectiveness in other applications.

## 4. Conclusions

The state-of-art deep network named RetinaNet was investigated to detect storm-drains and manholes in mobile mapping RGB images. RetinaNet was considered with a backbone composed of the ResNet-50 and the Resnet-101 models. THe approach revealed high accuracy in detecting both objects (with mAP higher than 90%). The RetinaNet method was suitable to detect storm-drains in terrestrial RGB imagery, and it outperformed the Faster R-CNN method.

In the future, the trained network will be able to be used to map entire urban catchments with the help of image-based mobile imagery to allow for the incorporation of manhole and storm-drain information into hydrologic and hydraulic modeling to better prevent and mitigate the impact of urban flood events. Other state-of-the-art methods should be proposed and tested to produce a more specific network, which is related to our previous work [21], that can handle this and similar tasks considering point annotation. We provide the labeled dataset used in this study and encourage future research to test the performance of new DL methods with this data. Because of the specific nature of this type of labeled data, it is usually not easily available, and hence it should benefit the training process for focused hydrological work in urban areas.

## Figures and Tables

**Table 1:** Distribution of the number of (#) images and classes on training, validation and testing data-sets for the division 72-12-12 and 66-15-19.

Division	Set	# Images (%)	# Manholes	# Storm-Drains
76-12-12	Train	226 (76%)	120	113
	Validation	35 (12%)	25	10
	Train + Validation	261 (88%)	145	123
	Test	36 (12%)	21	19
66-15-19	Train	198 (66%)	104	100
	Validation	44 (15%)	25	20
	Train + Validation	226 (81%)	129	120
	Test	55 (19%)	37	22

**Table 2:** Average precision values to AP50 and to classes manhole (${\mathrm{AP}}_{mh}$) and storm-drain (${\mathrm{AP}}_{sd}$).

Division	Method	Backbone	AP50(%)	${AP}_{mh}\left(\%\right)$	${AP}_{sd}\left(\%\right)$
76-12-12	Faster-RCNN	ResNet-50	88.30	95.24	71.93
		ResNet-101	86.32	95.24	71.15
	RetinaNet	ResNet-50	92.08	100.00	84.21
		ResNet-101	92.08	95.24	89.47
66-15-19	Faster-RCNN	ResNet-50	88.62	97.22	80.86
		ResNet-101	85.22	96.95	73.85
	RetinaNet	ResNet-50	88.85	94.01	84.42
		ResNet-101	89.69	94.22	85.93
